# SFRP4 Is a Potential Biomarker for the Prognosis and Immunotherapy for Gastric Cancer

**DOI:** 10.1155/2022/8829649

**Published:** 2022-07-05

**Authors:** Pengcheng Yu, Weiyang He, Yanqiang Zhang, Can Hu, Yue Wu, Yi Wang, Zhehan Bao, Yuhang Xia, Ruolan Zhang, Mengxuan Cao, Li Yuan, Xiangdong Cheng, Zhiyuan Xu

**Affiliations:** ^1^First Clinical Medical College of Zhejiang Chinese Medical University, Hangzhou 310053, China; ^2^Department of Gastrointestinal Surgery, Sichuan Cancer Hospital, Chengdu 610042, China; ^3^Cancer Hospital of the University of Chinese Academy of Sciences (Zhejiang Cancer Hospital), Institutes of Basic Medicine and Cancer, Chinese Academy of Sciences, Hangzhou 310022, China; ^4^Second Clinical Medical College of Zhejiang Chinese Medical University, Hangzhou 310053, China; ^5^Wenzhou Medical University, Wenzhou 325035, China; ^6^Key Laboratory of Prevention, Diagnosis and Therapy of Upper Gastrointestinal Cancer of Zhejiang Province, Hangzhou 310022, China; ^7^Zhejiang Provincial Research Center for Upper Gastrointestinal Tract Cancer, Zhejiang Cancer Hospital, Hangzhou 310022, China

## Abstract

**Purpose:**

Secreted frizzled-related protein 4 (SFRP4) is a member of the SFRP family, which functions as either a tumor suppressor or a prooncogenic factor in distinct tumor types. Our research aimed to explore the expression of SFRP4 in gastric cancer, its prognostic significance, and its relationship with immune cell infiltration.

**Materials and Methods:**

Gastric cancer and paracancerous tissue specimens from surgically resected gastric cancer patients were collected to construct tissue microarrays, and immunohistochemistry was used to detect the expression of SFRP4, PD-L1, CD3^+^*T*, CD4^+^*T*, and CD8^+^*T* in these microarrays. The differential expression of SFRP4 and its relationship with the immune microenvironment were evaluated using the TIMER and TISIDB databases. Finally, patient survival was assessed.

**Results:**

SFRP4 expression was elevated in gastric cancer tissues and linked to a poor prognosis (*P*=0.021). The 5-year survival rate for patients with high SFRP4 expression was only 39.81% but reached 60.02% for patients with low SFRP4 expression. Increased SFRP4 expression correlated with high CD8^+^ T-cell infiltration (*P*=0.015) and positive PD-L1 expression (*P*=0.036). High SFRP4 expression was an independent predictor of overall survival (*P*=0.024 in univariable analysis, *P*=0.011 in multivariable analysis). Using online databases, we found that SFRP4 expression was higher in gastric cancer tissues and substantially was associated with the immune microenvironment.

**Conclusion:**

SFRP4 is an oncogenic driver that can predict patient survival time in gastric cancer, as well as an important immune-related factor. SFRP4 may be important for guiding immunotherapy in gastric cancer patients.

## 1. Introduction

The cancer incidence and mortality from the International Agency for Research on Cancer (IARC) for 2020 are 19.3 million new cancer cases and 10 million cancer deaths globally. Gastric cancer is the fifth most frequent cancer in the world and the fourth leading cause of cancer-related deaths [1]. Despite recent breakthroughs in gastric cancer diagnosis and therapy, most patients are diagnosed with advanced gastric cancer due to the lack of obvious early symptoms and a poor diagnostic rate. The prognosis of gastric cancer remains dismal, with an overall 5-year survival rate of less than 40% [2]. As a result, finding effective biomarkers for the early identification of gastric cancer and development of new therapeutic techniques are critical.

Secretory frizzled-related protein 4 (SFRP4), a member of the secretory frizzled-related protein family, is a Wnt signaling inhibitor that plays a key role in cancer [3]. The SFRP4 gene is located on the short arm of chromosome 7 (7p14.1) and is made up of six 10.99 kb coding exons. SRP4 contains a cysteine-rich structural domain and is thought to be a tumor suppressor because of its similarity to the Wnt binding site [4]. SFRP4 has been found to prevent malignant tumor proliferation and metastasis [5]. However, Postovit and Vincent [6] found a trend toward higher SFRP4 expression during tumor growth, which may contradict the finding that SFRP4 acts as a tumor suppressor. Several studies have shown that SFRP4 expression is downregulated in tumors relative to surrounding normal tissues in esophageal, ovarian, liver, pancreatic, and breast cancers [7–11]. Other research has found that SFRP4 is overexpressed in colorectal, prostate, and thyroid malignancies compared to normal tissues [12–14]. The expression of SFRP4 and its role in gastric cancer development remains unknown.

The tumor immune microenvironment plays a key role in cancer development, and the complicated interactions between cancer cells and immune cells can either promote or hinder cancer progression. Immunotherapy has recently been shown to have immunomodulatory and antitumor benefits and can increase patients' susceptibility to chemotherapy by triggering an antitumor immune response. Considerable clinical research has shown that immunotherapy for gastric cancer has achieved superior results [15]. Antiprogrammed cell death-1 (PD-1) and programmed cell death ligand-1 (PD-L1) monoclonal antibodies are immune checkpoint inhibitors (ICIs) that can improve survival in gastric and other cancers [16]. Antibodies have been licensed for the treatment of metastatic and resistant gastric cancer patients [17, 18]. Nevertheless, ICI therapy has benefited only a limited percentage of gastric cancer patients [19]. As a result, new biomarkers must be investigated immediately to increase the number of people who can benefit from immunotherapy for gastric cancer. Based on the SFRP4, CPXM1, and COL5A1 genes, Chen et al. established an immune-related gene prognostic index (IRGPI) for head and neck squamous carcinoma, and a high IRGPI score was associated with higher infiltration of CD8^+^*T* and CD4^+^*T* cells and M1 macrophages [20]. SFRP4 may affect tumor growth by altering the tumor immune microenvironment and might be a potential therapeutic target for immunotherapy.

The goal of this research was to explore the expression of SFRP4 in gastric cancer, its clinical significance, and its relationship with the tumor immune microenvironment. We investigated SFRP4 expression and its effect on overall survival (OS) in 137 patients with gastric cancer. The influence of PD-L1 expression and *T*-cell infiltration on the OS of patients was examined, as well as the correlations between SFRP4 and PD-L1 expression and *T*-cell infiltration. In addition, we used the TIMER database to analyze the differential expression of SFRP4 in tumor tissues, the correlation between SFRP4 expression and *T*-cell infiltration, and the relationship between SFRP4 and the immune microenvironment based on the TISIDB database. This study finds that SFRP4 is an oncogenic driver that can predict patient survival time in gastric cancer, as well as an important immune-related factor. SFRP4 may be important for guiding immunotherapy in gastric cancer patients.

## 2. Materials and Methods

### 2.1. Patient Selection and Tissue Microarray Construction

A total of 137 patients with stomach cancer who were admitted to the Cancer Hospital of the University of Chinese Academy of Sciences (Zhejiang Cancer Hospital) between January 2013 and December 2017 were selected. The following inclusion criteria were used: (1) all of the samples had a pathological diagnosis of gastric cancer; (2) no antitumor treatment, such as chemoradiotherapy, biotherapy, or immunotherapy, had been administered before surgery; (3) the patients' medical records were complete. Patients with other types of malignant tumors in the past, patients who received antitumor therapy before surgery, and patients who had metastasis from other tumor types were excluded.

A total of 137 gastric cancer patients who underwent surgery in our hospital were included in this study. All of the surgically removed gastric cancer tissue and paracancerous tissue specimens of patients were collected, fixed in 4% paraformaldehyde, and paraffin-embedded. Tissue microarray technology was used to create paraffin tissue microarrays of gastric cancer tissues and paired paracancerous tissues, and immunohistochemistry was used to detect the expression of SFRP4, CD3^+^*T*, CD4^+^*T*, CD8^+^*T*, and PD-L1 in these microarrays. The clinicopathological information of these 137 patients was obtained retrospectively and included the following: patient's age; sex; history of smoking, drinking, and body weight; family history of gastric cancer; tumor location; Borrmann staging; Lauren staging; degree of differentiation; pathological type; tumor size; *T* stage; *N* stage; *M* stage; TNM stage; tumor markers; and 5-year survival rate. The TNM staging of gastric cancer referred to the eighth edition of the American Joint Committee on Cancer (AJCC) staging guidelines.

## 3. Immunohistochemical Evaluation

The collected gastric cancer tissue specimens and paraneoplastic tissue specimens were formalin-fixed and paraffin-embedded. Representative gastric cancer samples and paraneoplastic tissues were chosen for tissue microarrays after independent screening by two pathologists. After dewaxing and rinsing the sections with distilled water, antigen retrieval was performed, and the sections were washed with PBS for 5 minutes three times. Next, SFRP4, CD3^+^*T*, CD4^+^*T*, CD8^+^*T*, and PD-L1 antibodies (SFRP4: Proteintech, 15328-1-AP, dilution ratio 1 : 300; CD3^+^*T*: Abcam, ab16669, dilution ratio 1 : 200; CD4^+^*T*: Abcam, ab133616, dilution ratio 1 : 200; CD8^+^*T*: Abcam, ab17147, dilution ratio 1 : 200; PD-L1: DAKO/Agilent, SK006, dilution ratio 1 : 50) were added, incubated overnight at 4°C; then slides were washed for 5 minutes three times in PBS. Then, the appropriate secondary goat anti-rabbit IgG H&L (PV-9003, ZSGB-BIO Corp., Shanghai, China; dilution ratio 1 : 1000) or goat anti-mouse IgG H&L (ISH-7003, ZSGB-BIO Corp., Shanghai, China; dilution ratio 1 : 500) antibody was added to the tissue microarray, incubated for 30 minutes, and washed with PBS for 5 minutes three times. DAB color development and hematoxylin staining of cell nuclei were performed with a DAB color development kit (ZLI-9065, ZSGB-BIO Corp., Shanghai, China). Finally, the tissue microarray was dehydrated and closed with neutral gel closure (G8590, Solarbio, Beijing, China).

The number of CD3^+^*T*, CD4^+^*T*, and CD8^+^*T* cells on the microarrays was counted [21]. According to the median number of stained cells, the patients were split into high and low expression groups. The combined positive score (CPS), which was calculated as CPS = [number of PD-L1 positive cells (tumor cells, lymphocytes, and macrophages)/total tumor cells] × 100, was used to express PD-L1 expression. CPS ≥10 was scored as positive [22].

The intensity of SFRP4 expression was measured using the H-scoring system, which uses formula H-score =  (IS × AP), where IS represents the intensity of staining and AP represents the percentage of positively stained cells. IS was determined by the staining of the cells: 0 for no staining; 1 for weak staining; 2 for moderate staining; and 3 for strong staining. The percent of AP-stained cells was scored as follows: 0% was scored as 0; 1–25% was scored as 1; 26–50% was scored as 2; 51–75% was scored as 3; and 76–100% was scored as 4. The patients were classified into two groups based on the median of their scores: high SFRP4 expression and low SFRP4 expression groups. The above results were interpreted by two associate senior-level pathologists.

### 3.1. Database Analysis

The TIMER [23] database was used to perform differential expression analysis. The TIMER and TISIDB [24] databases were used to investigate the link between SFRP4 and the immunological microenvironment.

### 3.2. Statistical Analysis

The data were analyzed using SPSS 26.0 statistical software, and graphing was performed using GraphPad Prism 9 software. The counts are reported as values and percentages, and the measurements were examined using the chi-square test and Fisher's exact test. The Kaplan-Meier method was used for survival analysis, and Cox regression was used for univariate multifactorial risk assessment. A statistically significant difference was defined as *P* < 0.05.

## 4. Results

### 4.1. General Information

A retrospective cohort study was conducted on 137 gastric cancer patients, including 97 (70.80%) males and 40 (29.20%) females; the median age was 61 (28–86) years. There were 43 (31.39%) patients with tumors in the proximal stomach, 85 (62.04%) patients with tumors in the distal stomach, and 9 (6.57%) patients with tumors in the whole stomach. There were 69 (50.36%) patients with the intestinal type of Lauren typing, 18 (13.14%) patients with the mixed Lauren type, and 50 (36.50%) patients with the diffuse Lauren type. One (0.73%) patient had TNM stage I, 17 (12.41%) patients had stage II, 106 (77.37%) patients had stage III, and 13 (9.49%) patients had stage IV. The median OS was 39 (2–92) months; a total of 73 (53.28%) patients died during the follow-up period. The remaining detailed clinicopathological characteristics of the patients are shown in Table 1.

### 4.2. SFRP4 Is Overexpressed in Gastric Cancer and Is Correlated with the Depth of Tumor Infiltration

**Figure 1:**
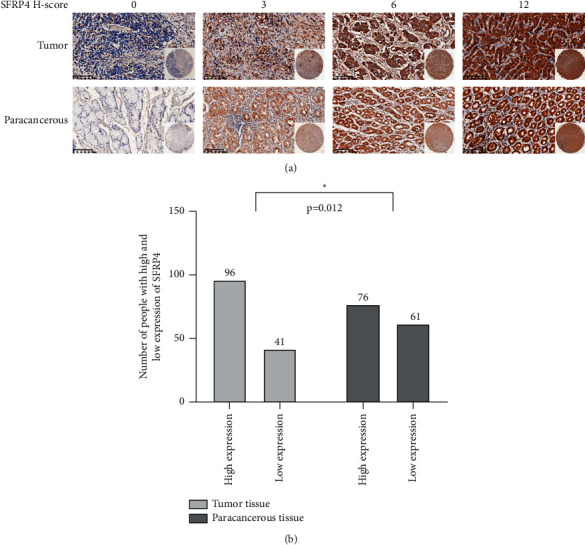
SFRP4 is overexpressed in gastric cancer tissues. (a) SFRP4 expression in representative gastric tumor and paracancerous tissues (immunohistochemical staining, x20). (b) SFRP4 expression differences in tumor and paracancerous tissues (*n* = 137).

**Figure 2:**
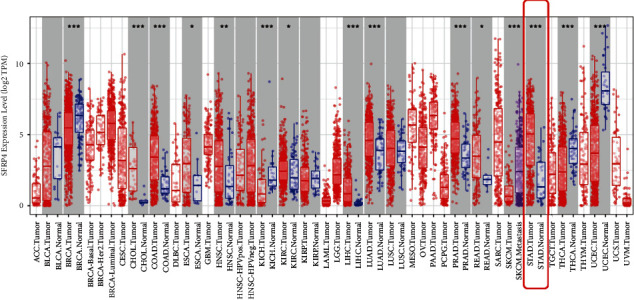
TIMER database showing that SFRP4 expression differs in various cancers (M (IQR), Wilcoxon test).

SFRP4 was found to be expressed in the cell membrane and cytoplasm in both gastric cancer and paraneoplastic tissues but was minimally expressed in the nucleus (Figure 1(a)). In these 137 patients, high expression of SFRP4 was found in 96 (70.07%) cancer tissues and 76 (55.47%) paracancerous tissues (Table 2), and SFRP4 expression was considerably elevated in cancer tissues compared to paracancerous tissues (*P*=0.012) (Figure 1(b)). The degree of SFRP4 expression was positively correlated with the depth of tumor infiltration (*P*=0.025), and no significant relationship between SFRP4 expressions in gastric cancer tissues was found for age, sex, body weight, family history of gastric cancer, tumor location, Borrmann staging, Lauren staging, or degree of differentiation. A significant relationship was found between SFRP4 and clinicopathological characteristics such as pathological type, tumor size, N stage, M stage, and TNM stage (Table 3). To validate further the expression of SFRP4 in gastric cancer tissues, the TIMER database was used to assess the difference in SFRP4 expression between malignant tumors and their equivalent normal tissues. That assessment matched our findings (Figure 2) that SFRP4 was overexpressed in STAD (stomach adenocarcinoma) tissues.

### 4.3. Positive PD-L1 Expression and High CD8^+^*T*-Cell Infiltration Are Positively Linked to High SFRP4 Expression

**Figure 3:**
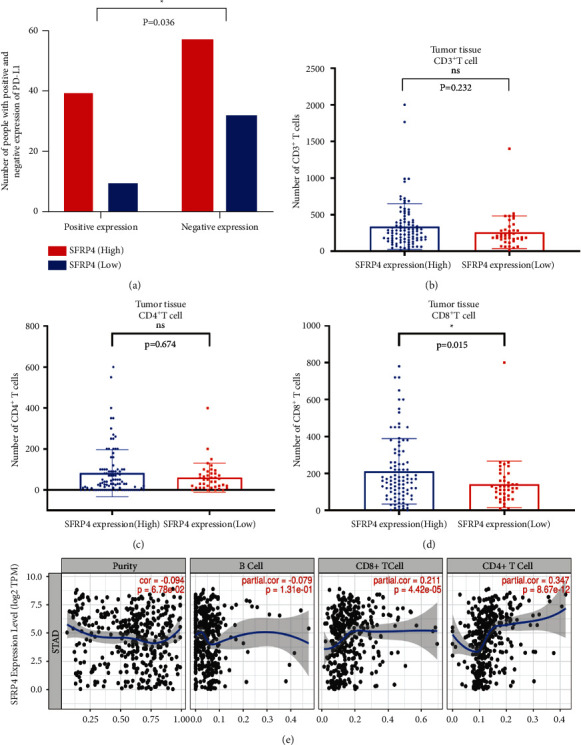
Association of SFRP4 expression with PD-L1 and tumor-infiltrating lymphocytes in gastric cancer. (a) Association between PD-L1 expression and SFRP4 expression in gastric cancer. (b) Correlation between CD3^+^*T* cells and SFRP4 expression in gastric cancer. (c) Association between CD4^+^*T* cells and SFRP4 expression in gastric cancer. (d) Association between CD8^+^*T* cells and SFRP4 expression in gastric cancer. (e) Correlation between SFRP4 expression and immune cells in gastric adenocarcinoma in the TIMER database (purity-corrected partial Spearman's rho value and statistical significance).

The chi-square test was used to examine the relationship between SFRP4 expression and PD-L1 expression in gastric cancer tissues, and high SFRP4 expression was determined to be positively linked with the degree of PD-L1 expression (Figure 3(a)). The rank-sum test was used to examine the relationship between SFRP4 expression and *T*-cell infiltration. CD8^+^*T*-cell infiltration was shown to be greater in gastric cancer tissues with elevated SFRP4 expression (*P*=0.015) (Figure 3(b)). Between the high and low SFRP4 expression groups, the degree of CD3^+^*T* and CD4^+^*T*-cell infiltration did not differ substantially (Figures 3(c) and 3(d)). The TIMER database was used to investigate the relationship between SFRP4 expression and immune cell infiltration in gastric cancer. SFRP4 expression was found to associate with CD8^+^*T*-cell (partial correlation = 0.211, *P*=4.42*e* − 05) and CD4^+^*T*-cell (partial correlation = 0.347, *P*=8.67*e* − 12) expression. Based on our findings and database analysis, these results indicate that SFRP4 can regulate the immune microenvironment of gastric cancer (Figure 3(e)).

### 4.4. SFRP4 Expression Is Correlated with the Immune Microenvironment

**Figure 4:**
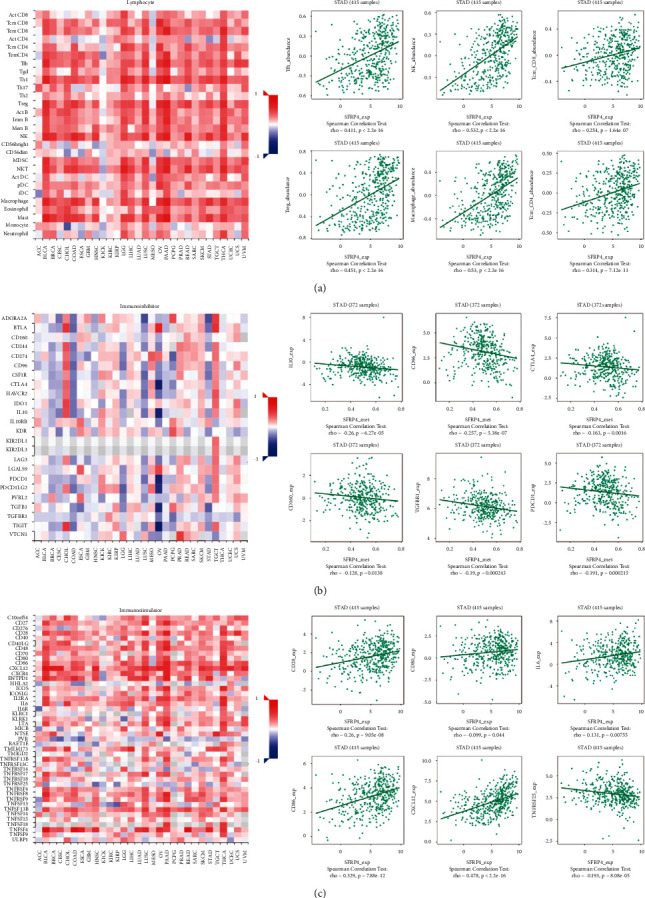
Association between SFRP4 expression and immune components, including lymphocytes and immunomodulators in patients with stomach adenocarcinoma. (a) Correlation between SFRP4 expression levels and lymphocytes. (b) Relationship between SFRP4 expression levels and immunoinhibitors. (c) Correlation between SFRP4 expression levels and immunostimulators.

**Figure 5:**
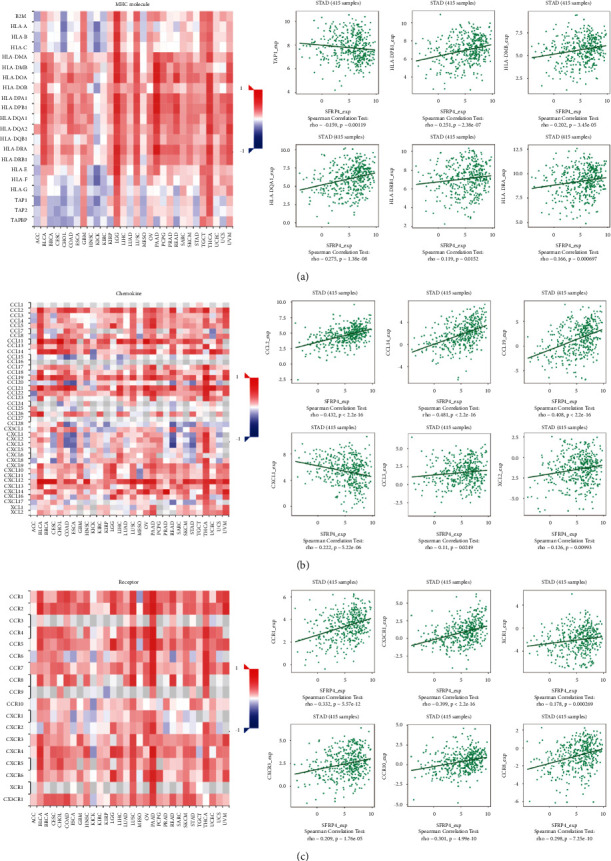
Association between SFRP4 expression and immune components, including MHC molecules, chemokines, and receptors in patients with stomach adenocarcinoma. (a) Correlation between SFRP4 expression levels and MHC molecules. (b) Relationship between SFRP4 expression levels and chemokines. (c) Correlation between SFRP4 expression levels and receptors.

To further explore whether there is an association between SFRP4 expression and the immune microenvironment, we analyzed the relationship between SFRP4 expression levels and immune components in gastric adenocarcinoma patients using the TISIDB database. We first investigated the relationship between the infiltration abundance of lymphocytes in the tumor and SFRP4 expression levels. Here, we found that SFRP4 expression levels correlated with Tfh cells (rho (Spearman's Rank Correlation Coefficient) = 0.411, *P* < 2.2*e* − 16), NK cells (rho = 0.532, *P* < 2.2*e* − 16), Tcm_CD8 cells (rho = 0.254, *P* < 1.64*e* − 07), Treg cells (rho = 0.451, *P* < 2.2*e* − 16), macrophage cells (rho = 0.53, *P* < 2.2*e* − 16), and Tcm_CD4 cells (rho = 0.314, *P* < 7.12*e* − 11) (Figure 4(a)). We next evaluated the relationships between three immunomodulators (immunosuppressive molecules, immune agonists, and major histocompatibility complex molecules) and the expression of SFRP4. SFRP4 expression levels correlated with the immunosuppressive molecules IL10, CD96, CD160, CTLA4, TGFBR1, and PDCD1 (Figure 4(b)), and, with immune agonist molecules, including CD28, CD86, CXCL12, CD80, IL6, and TNFRSF25 (Figure 4(c)). SFRP4 expression also correlated with the major histocompatibility complex molecules TAP1, HLA-DPB1, HLA-DMB, HLA-DQA1, HLA-DRB1, and HLA-DRA (Figure 5(a)). Finally, we analyzed the relationship between SFRP4 expression and chemokines and receptors. Expression of chemokines, including CCL2, CCL14, CCL19, CXCL1, CCL3, and XCL2, correlated with SFRP4 expression (Figure 5(b)). Receptor expression, including CCR1, CX3CR1, XCR1, CXCR3, CCR10, and CCRB, also correlated with SFRP4 expression (Figure 5(c)). These results further confirmed that SFRP4 plays an important regulatory role in the immune microenvironment of gastric cancer tumors and may be a key critical target for gastric cancer immunotherapy.

### 4.5. SFRP4 Is an Independent Prognostic Marker for Gastric Cancer and, When Paired with CD8^+^*T* Cells, Can Improve Gastric Cancer Prognosis

**Figure 6:**
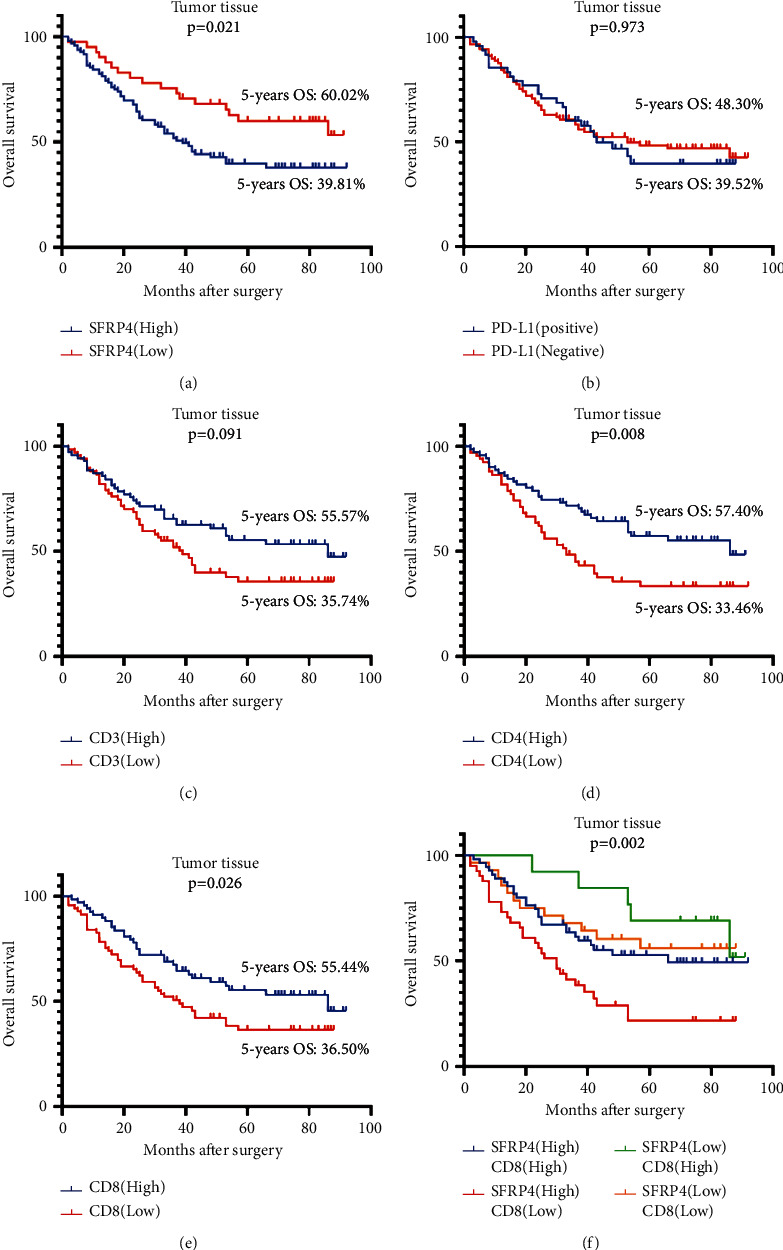
In gastric cancer, SFRP4 is an independent prognostic factor, and SFRP4 paired with CD8^+^*T* cells can better predict prognosis. (a) Kaplan-Meier survival curves for OS in tumor tissues based on SFRP4 expression. (b) Kaplan-Meier survival curves for OS based on tumor tissue PD-L1 expression. (c) Kaplan-Meier survival curves for OS in tumor tissues based on CD3+ *T*-cell expression. (d) Kaplan-Meier survival curves for OS in tumor tissues based on CD4+ T-cell expression. (e) Kaplan-Meier survival curves for OS in tumor tissues based on CD8+ *T*-cell expression. (f) Kaplan-Meier survival curves for OS based on SFRP4 expression in tumor tissues in combination with CD8^+^*T* expression.

The expression of SFRP4, PD-L1, CD3^+^*T*, CD4^+^*T*, and CD8^+^*T* in the tumors was used to determine OS using Kaplan-Meier analysis. The group with high SFRP4 expression in gastric cancer tissues had a poorer 5-year OS rate (*P*=0.021), where the 5-year OS rates in the low and high SFRP4 expression groups were 60.02% and 39.81%, respectively (Figure 6(a)). When compared to the low expression groups, the high CD4^+^*T* and CD8^+^ T-cell infiltration groups had a better OS (*P*=0.008 and *P*=0.026, resp.) (Figures 6(d) and 6(e)). No significant difference was found in OS between the PD-L1-positive and PD-L1-negative groups (*P*=0.973) or between the groups with high and low CD3^+^*T* expression (*P*=0.091) (Figures 6(b) and 6(c)). In comparison to the other groups, the group that had low SFRP4 expression with high infiltration of CD8^+^*T* cells had the best prognosis, with a 5-year OS rate of 69.23% (Figure 6(f)).

To examine prognostic values, we analyzed various clinicopathological characteristics using Cox proportional hazards regression models. We found that high tumor SFRP4 expression (*P*=0.024), family history of gastric cancer (*P*=0.005), high CD4 expression (*P*=0.009), and high CD8^+^*T* expression (*P*=0.048) were risk or protective factors for OS in patients in univariate analysis (Table 4). High SFRP4 expression (*P*=0.011) and having a family history of gastric cancer (*P*=0.011) were found to be independent predictors of OS in patients with gastric cancer in multivariate analysis. In multivariate analysis, other clinicopathological characteristics, such as high CD4 (*P*=0.708) and CD8 (*P*=0.060) expression, did not show significant differences (Table 5). In both univariate and multivariate analyses, SFRP4 was found to be an independent prognostic factor for gastric cancer.

## 5. Discussion

The most common treatment technique for stomach cancer is surgical resection. However, due to the limitations of tumor stage and tumor molecular typing, it is difficult to achieve radical resection with surgical treatment, which can easily lead to tumor recurrence or metastasis after surgery. With the rapid advancement of tumor immunology in recent years, immunotherapy has emerged as a new treatment option for many cancers, improving the survival rate of patients with advanced tumors. ICIs, such as monoclonal antibodies against PD-1 or PD-L1, have emerged as promising novel approaches to cancer treatment, including gastric cancer. However, new immunotherapy targets have yet to be discovered, and the number of gastric cancer patients who benefit from immunotherapy remains small. The goal of this research was to determine the significance of SFRP4 and the immune microenvironment in the treatment and prognosis of gastric cancer.

SFRP1, SFRP2, SFRP3, SFRP4, and SFRP5 are members of the SFRP family of glycoproteins. SFRPs contain two major structural domains that function independently of each other [25]. The C-terminal domain contains a netrin-like structural domain (NLD). The N-terminal domain has a 120-amino acid cysteine-rich structural domain (CRD), which includes a conserved 10-amino acid cysteine-rich region with a strong sequence similarity to the CRD region of the Wnt receptor Frizzled (Fz) protein [4]. Recent studies found that NLD is involved in cell apoptosis and the CRD is required for angiogenesis suppression. Additionally, both CRD and NLD can raise intracellular calcium levels and activate the Wnt/Ca^2+^ signaling pathway [26]. The Wnt signaling pathway plays a vital role in cell survival, proliferation, and polarity [3]. Wnt signaling is thought to be a significant element in tumor growth and contribute to carcinogenesis in general. However, SFRPs in the Wnt signaling pathway may have a bidirectional regulatory mechanism of action [27]. As a result, the association between SFRP4 expression in tumors and carcinogenesis development is unknown.

Previous research has found that SFRP4 is highly expressed in colorectal cancer patients and that individuals with high SFRP4 expression have a worse prognosis than those with low SFRP4 expression [28]. SFRP4 expression was considerably downregulated in pancreatic cancer tissues, and patients with low SFRP4 expression had a better prognosis than those with high SFRP4 expression [9]. These observations suggest that SFRP4 may play an important role in tumor formation. Using tissue microarray technology, we discovered that SFRP4 expression was significantly elevated in gastric cancer tissues compared to paracancerous tissues and tumor infiltration was deeper in patients with high SFRP4 expression. Our further survival study revealed that elevated SFRP4 expression in gastric cancer is associated with a worse prognosis, with a 5-year OS rate of 39.81%. We also found that SFRP4 expression status was an independent predictive factor for gastric cancer patients in univariate and multifactorial models. Therefore, the elevation in SFRP4 expression occurs during gastric carcinogenesis and development, and high SFRP4 expression indicates a poor prognosis.

By evaluating the association between SFRP4 expression and PD-L1 expression, we discovered a positive correlation between high SFRP4 expression and positive PD-L1 expression. High SFRP4 expression in gastric cancer patients likely corresponds with positive PD-L1 expression, resulting in a poor prognosis [29, 30]. PD-1 and its ligand PD-L1 reduce cytotoxic T-cell responses in immunological responses, resulting in tumor cell immune evasion and poor prognosis. Increased PD-L1 expression was found to be positively linked with high CD8^+^ T-cell infiltration in a pan-cancer research study [31]. PD-L1 positivity in gastric cancer was substantially linked with CD8^+^ T-cell infiltration in another investigation [32]. Based on these findings, we postulate that SFRP4 regulates the immunological milieu via PD-L1 expression, influencing patient prognosis.

SFRP4 expression correlated with a significant infiltration of CD8^+^ T cells and CD4^+^*T* cells in head and neck squamous cell cancer [20]. High SFRP4 expression is favorably linked with CD8^+^ T-cell infiltration, according to our findings. To validate this result, we analyzed the TIMER database to verify the correlation between SFRP4 expression and immune cell infiltration in STAD. The results revealed a significant positive correlation between SFRP4 expression and CD8^+^*T* and CD4^+^ T cells. Survival analysis showed that high CD4^+^*T* and CD8^+^*T*-cell infiltration was associated with better OS than low infiltration. Increased CD8^+^*T*-cell infiltration in tumor tissue was adversely linked with tumor recurrence in one investigation [33]. In gastric cancer, the density of CD8^+^*T*-cell infiltration is an independent predictor of clinical outcome [34]. Using the TISIDB database to evaluate the link between SFRP4 expression and the immune milieu, we found a clear association between SFRP4 expression levels and lymphocytes, immunomodulators, and chemokines in gastric cancer patients. Based on these findings, we believe that SFRP4 interacts with immune cell infiltration throughout the progression of gastric cancer, influencing the immune microenvironment. To explain the simultaneous accumulation of many immune cells, we propose that CD8^+^*T* lymphocyte infiltration recruits additional immune cells by stimulating the release of specific signals on the cell surface, thereby controlling the involvement of immune cells throughout tumor formation. Furthermore, patients with low SFRP4 expression and high CD8^+^*T*-cell infiltration had the greatest survival benefit, which could aid in the selection of an appropriate immunotherapy strategy in the future.

Our results indicate that SFRP4 expression is upregulated in gastric cancer tissues. SFRP4 is an independent prognostic factor and is significantly associated with poor prognosis in gastric cancer patients. Additionally, high SFRP4 expression is positively correlated with positive PD-L1 expression and high CD8^+^*T* infiltration. Furthermore, online database mining revealed that several different lymphocytes, immunomodulators, and chemokines in gastric cancer tissues were substantially linked with high SFRP4 expression. SFRP4 is a potential biomarker for guiding immunotherapy because it can represent the condition of immune microenvironment.

## Figures and Tables

**Table 1:** Clinicopathological characteristics of the patient cohort (*n * = 137).

Variables	Patients, % (n)
Expression of SFRP4	
High	70.07 (96)
Low	29.93 (41)
Age (year)	
>60	53.28 (73)
≤55	46.72 (64)
Sex	
Female	29.20 (40)
Male	70.80 (97)
Smoking	
Yes	36.50 (50)
No	63.50 (87)
Drinking	
Yes	24.09 (33)
No	75.91 (104)
Weight loss	
Yes	38.69 (53)
No	60.58 (83)
Unknown	0.73 (1)
Family history	
Yes	14.60 (20)
No	85.40 (117)
Tumor location	
Proximal gastric cancer	31.39 (43)
Distal gastric cancer	62.04 (85)
Total stomach	6.57 (9)
Borrmann type	
I/II	56.20 (77)
III/IV	42.34 (58)
Unknown	1.46 (2)
Lauren type	
Intestinal	50.36 (69)
Diffuse	36.50 (50)
Mixed	13.14 (18)
Grade of differentiation	
Poor/well	44.53 (61)
Moderate/moderate-poor	51.82 (71)
Unknown	3.65 (5)
Pathological type	
Signet ring cell carcinoma/mucinous adenocarcinoma	10.22 (14)
Adenocarcinoma	89.78 (123)
Tumor size (cm)	
≥5 cm	64.96 (89)
<5 cm	32.85 (45)
Unknown	2.19 (3)
T stage	
T1/T2	5.11 (7)
T3/T4	94.89 (130)
N stage	
N0	7.30 (10)
N1/N2/N3	92.70 (127)
M stage	
M0	90.51 (124)
M1	9.49 (13)
TNM stage	
I	0.73 (1)
II	12.41 (17)
III	77.37 (106)
IV	9.49 (13)
Nerve invasion	
Positive	75.18 (103)
Negative	24.82 (34)
Vascular invasion	
Positive	58.39 (80)
Negative	41.61 (57)
AFP (ng/ml)	
>8.1	5.84 (8)
≤8.1	92.70 (127)
Unknown	1.46 (2)
CA19-9 (U/ml)	
>37	33.58 (46)
≤37	64.69 (90)
Unknown	0.73 (1)
PD-L1	
Positive	35.04 (48)
Negative	64.96 (89)
CD3	
High	51.09 (70)
Low	48.91 (67)
CD4	
High	51.82 (71)
Low	48.18 (66)
CD8	
High	49.64 (68)
Low	50.36 (69)

**Table 2:** Differential expression of SFRP4 in gastric cancer and paracancerous tissues.

Variables	*N*	Expression of SFRP4			*χ* ^2^	*P* value^a^
		High	Low	High rate		
Tumor tissue	137	96	41	70.07%	6.247	0.012^∗^
Paracancerous tissue	137	76	61	55.47%		

^a^^*∗*^Statistically significant (*P* < 0.05).

**Table 3:** Association of SFRP4 expression with clinicopathological characteristics of patients with gastric cancer (*n* = 137).

Variables	Expression		Total	High rate	*χ* ^2^	*P* value^b^
	High	Low				
Age (year)						
>60	52	21	73	71.23%	0.100	0.752
≤60	44	20	64	68.75%		
Sex						
Female	30	10	40	75.00%	0.654	0.419
Male	66	31	97	68.04%		
Smoking						
Yes	33	17	50	66.00%	0.623	0.430
No	63	24	87	72.41%		
Drinking						
Yes	20	13	33	60.61%	1.858	0.173
No	76	28	104	73.08%		
Weight loss						
Yes	38	15	53	71.70%	0.140	0.708
No	57	26	83	68.67%		
Unknown	1	0	1	100%		
Family history						
Yes	12	8	20	60.00%	1.133	0.287
No	84	33	117	71.79%		
Tumor location						
Proximal gastric cancer	31	12	43	72.09%	1.971	0.373
Distal gastric cancer	57	28	85	67.06%		
Total stomach	8	1	9	88.89%		
Borrmann type						
I/II	54	23	77	70.13%	0.005	0.944
III/IV	41	17	58	70.69%		
Unknown	1	1	2	50%		
Lauren type						
Intestinal	45	24	69	65.22%	1.653	0.438
Diffuse	38	12	50	76.00%		
Mixed	13	5	18	72.22%		
Grade of differentiation						
Poor/well	40	21	61	65.57%	0.913	0.339
Moderate/moderate-poor	52	19	71	73.24%		
Unknown	4	1	5	80%		
Pathological type						
Signet ring cell carcinoma/mucinous adenocarcinoma	8	6	14	57.14%	0.355	0.207
Adenocarcinoma	88	35	123	71.54%		
Tumor size (cm)						
≥5 cm	66	23	89	74.15%	2.033	0.154
<5 cm	28	17	45	62.22%		
Unknown	2	1	3	66.67%		
*T* stage						
T1/T2	2	5	7	28.57%	0.025	0.025
T3/T4	94	36	130	72.31%		
*N* stage						
N0	9	1	10	90.00%	0.281	0.141
N1/N2/N3	87	40	127	68.50%		
*M* stage						
M0	85	39	124	68.55%	0.343	0.191
M1	11	2	13	84.62%		
TNM stage						
I/II/III	85	39	124	68.55%	0.343	0.191
IV	11	2	13	84.62%		
Nerve invasion						
Positive	72	31	103	69.90%	0.006	0.940
Negative	24	10	34	70.59%		
Vascular invasion						
Positive	56	24	80	70.00%	<0.001	0.982
Negative	40	17	57	70.18%		
AFP (ng/ml)						
>8.1	6	2	8	75.00%	1.000	0.560
≤8.1	89	38	127	70.08%		
Unknown	1	1	2	50.00%		
CA19-9 (U/ml)						
>37	36	10	46	78.26%	2.333	0.127
≤37	59	31	90	65.56%		
Unknown	1	0	1	100.00%		

^b^Statistically significant (*P* < 0.05).

**Table 4:** Univariate analysis of prognostic parameters for survival in gastric cancer patients.

Variable	Univariable analysis	
	HR (95% CI)	*P* value^c^
SFRP4 (high vs. low)	1.875 (1.085–3.240)	0.024
Age (years) (≥65 vs. <65)	0.806 (0.509–1.276)	0.358
Sex (female vs. male)	1.245 (0.759–2.040)	0.385
Smoking (yes vs. no)	1.191 (0.743–1.911)	0.468
Drinking (yes vs. no)	1.179 (0.699–1.990)	0.537
Weight loss (yes vs. no)	1.226 (0.771–1.951)	0.390
Family history (yes vs. no)	2.258 (1.274–4.004)	0.005
Tumor location		
Total stomach (ref)	Ref	
Distal gastric cancer	0.355 (0.159–0.793)	0.011
Proximal gastric cancer	0.347 (0.148–0.818)	0.015
Borrmann type (I/II vs. III/IV)	0.587 (0.369–0.933)	0.024
Lauren type		
Mixed (ref)	Ref	
Diffuse	0.660(0.334–1.305)	0.232
Intestinal	0.494(0.255–0.956)	0.036
Grade of differentiation (poor/well vs. moderate-poor/moderate)	0.957(0.597–1.533)	0.854
Pathological type (signet ring cell carcinoma/mucinous adenocarcinoma vs. adenocarcinoma)	0.680 (0.295–1.570)	0.367
Tumor size (cm) (≥5 cm vs. <5 cm)	1.358 (0.816–2.263)	0.239
T stage (T1/T2 vs. T3/T4)	0.369 (0.090–1.507)	0.165
N stage (N0 vs. N1/n2/n3)	0.655 (0.205–2.092)	0.475
M stage (M0 vs. M1)	0.266 (0.141–0.503)	<0.001
TNM stage (I/II/III vs. IV)	0.266 (0.141–0.503)	<0.001
Nerve invasion (positive vs. negative)	1.583 (0.884–2.838)	0.123
Vascular invasion (positive vs. negative)	2.046 (1.240–3.377)	0.005
AFP (ng/ml) (>8.1 vs. ≤8.1)	1.243 (0.538–2.870)	0.610
CA19-9 (U/ml) (>37 vs. ≤37)	1.469 (0.929–2.324)	0.222
PD-L1 (positive vs. negative)	1.071 (0.662–1.733)	0.779
CD3 (high vs. low)	0.646 (0.406–1.028)	0.065
CD4 (high vs. low)	0.538 (0.337–0.858)	0.009
CD8 (high vs. low)	0.625 (0.393–0.995)	0.048

^c^Statistically significant (*P* < 0.05).

**Table 5:** Multivariable analysis of prognostic parameters for survival in gastric cancer patients.

Variable	Multivariable analysis	
	HR (95% CI)	*P* value^d^
SFRP4 (high vs. low)	2.174 (1.196–3.951)	0.011
Family history (yes vs. no)	2.318 (1.217–4.414)	0.011
Tumor location (proximal gastric cancer vs. total stomach)	0.542 (0.208–1.413)	0.210
Tumor location (distal gastric cancer vs. total stomach)	0.412 (0.154–1.103)	0.078
Borrmann type (I/II vs. III/IV)	0.694 (0.423–1.136)	0.146
Lauren type (intestinal vs. mixed)	0.701 (0.341–1.440)	0.333
M stage (M0 vs. M1)	0.501 (0.249–1.008)	0.053
TNM stage (I/II/III vs. IV)	0.501 (0.249–1.008)	0.053
Vascular invasion (positive vs. negative)	1.549 (0.897–2.674)	0.116
CD4 (high vs. low)	0.896 (0.504–1.593)	0.708
CD8 (high vs. low)	0.594 (0.345–1.022)	0.060

^d^Statistically significant (*P* < 0.05).

## Data Availability

The datasets generated during and/or analyzed during the current study are not publicly available due to hospital policy but are available from the corresponding author on reasonable request.
